# A non-negative spike-and-slab lasso generalized linear stacking prediction modeling method for high-dimensional omics data

**DOI:** 10.1186/s12859-024-05741-6

**Published:** 2024-03-20

**Authors:** Junjie Shen, Shuo Wang, Yongfei Dong, Hao Sun, Xichao Wang, Zaixiang Tang

**Affiliations:** 1 Department of Biostatistics, School of Public Health, Jiangsu Key Laboratory of Preventive and Translational Medicine for Geriatric Diseases, MOE Key Laboratory of Geriatric Diseases and Immunology, Suzhou Medical College of Soochow University, No. 199 Renai Road, Suzhou, 215123 Jiangsu People’s Republic of China; 2 Institute of Medical Biometry and Statistics, Faculty of Medicine and Medical Center, University of Freiburg, 79085 Freiburg, Germany

**Keywords:** Stacking Bayesian method, Non-negative spike-and-slab prior, Omics segmentation

## Abstract

**Background:**

High-dimensional omics data are increasingly utilized in clinical and public health research for disease risk prediction. Many previous sparse methods have been proposed that using prior knowledge, e.g., biological group structure information, to guide the model-building process. However, these methods are still based on a single model, offen leading to overconfident inferences and inferior generalization.

**Results:**

We proposed a novel stacking strategy based on a non-negative spike-and-slab Lasso (nsslasso) generalized linear model (GLM) for disease risk prediction in the context of high-dimensional omics data. Briefly, we used prior biological knowledge to segment omics data into a set of sub-data. Each sub-model was trained separately using the features from the group via a proper base learner. Then, the predictions of sub-models were ensembled by a super learner using nsslasso GLM. The proposed method was compared to several competitors, such as the Lasso, grlasso, and gsslasso, using simulated data and two open-access breast cancer data. As a result, the proposed method showed robustly superior prediction performance to the optimal single-model method in high-noise simulated data and real-world data. Furthermore, compared to the traditional stacking method, the proposed nsslasso stacking method can efficiently handle redundant sub-models and identify important sub-models.

**Conclusions:**

The proposed nsslasso method demonstrated favorable predictive accuracy, stability, and biological interpretability. Additionally, the proposed method can also be used to detect new biomarkers and key group structures.

**Supplementary Information:**

The online version contains supplementary material available at 10.1186/s12859-024-05741-6.

## Background

Using high-dimensional omics data to build disease risk prediction models is a hotspot in research of clinical and public health fields. For example, a persistent challenge in cancer treatment is the heterogeneity of prognostic between patients [1], which is largely determined by the individual’s genetic and molecular makeup [2]. Precision medicine aims to use information at the high-dimensional molecular level and mathematical models, to achieve more accurate diagnosis, personalized medical care, and reliable prognosis prediction [3]. However, variable selection problem often exists in high dimensional data [4].

An ideal model should own interpretability, such that the end-users can comprehend and utilize it effectively [5]. Sparse models such as the Lasso and Elastic net, are considered to be more interpretable since they emphasize the limited number of important features that contribute more to prediction [6]. Extensions in a similar spirit include the smoothly clipped absolute deviation (SCAD) and the minimax concave penalty (MCP), which were introduced by [7] and [8], respectively. However, these methods are modeled on single-level information, such as gene expression data, which may ignore the interaction and higher-level linkage between variables. Network-based regularization method is an alternative approach, in which gene–gene interactions are utilized as extra regularization terms [9]. Besides, considering that modeling using only gene-level information will yield unstable results, building a model from the angle of a higher level of prior information (e.g. biological functions involved in disease mechanisms) is preferable [10, 11]. For example, carcinogenesis is a complex biological process regulated by multiple genes in various pathways, therefore, employing such pathway information in modeling can make better use of prior biological knowledge and is a closer mimic of tumor progression [12–16]. In this light, many methods, such as group Lasso (grlasso), group SCAD (grSCAD) and composite MCP (cMCP), have been proposed that enable the information of group structure to be integrated into model building procedure and can achieve sparsity at the group level or bi-level [17–19]. Group structure information can also be incorporated into predictive modeling through a two-step approach [11]. For instance, multi-layer group-Lasso (MLGL) sequentially apples hierarchical clustering and group Lasso to identify data-driven group structures and construct predictive models [20]. Ordered homogeneity pursuit Lasso (OHPL) first reduces the whole data to a set of variables that are representatives of group structures and then employs lasso to fit these dimension-reduced data [21]. A similar process was reported in an earlier study conducted by Chen and Wang [14, 22]. Compared to the data-driven group information, group information based on biological knowledge would be more robust to outlying samples [23]. Wei et al. introduced a pathway-based procedure for the integration of genomic data [16]. They used nonparametric models to fit the genes in each pathway and performed gradient descent boosting to combine the “pathway activities” additively. Zhang et al. proposed to integrate the risk scores derived from pathways using a Bayesian hierarchical Cox model to make cancer survival prediction [24]. Most of the aforementioned methods are single-model-based (SMB), which may result in inferior generalization ability in different data [25], while others employ a naive idea of ensemble learning.

The ensemble learning method is a general statistical practice that considers the predictions of multiple algorithms or models simultaneously [26–29]. By leveraging the strengths of varied models, ensemble methods often yield more robust and accurate predictions than using a single model [30]. Owing to these favorable properties, ensemble methods have gained increasing attention in the last two decades [31–34]. A popular ensemble learning method is “model stacking” or “stacked generalization” [26], to which we refer as “stacking” hereinafter. Stacking is usually a two-layer construction: in the first layer, a set of sub-models (base learners) are constructed and their predictions are harvested; in the second layer, a meta-model (super learner) is fitted by learning the predictions of sub-models. The predictions of sub-models are usually generated through a *K*-fold cross-validation (CV) manner to reduce overfitting [35]. Breiman stated in his study that “stacking never does worse than selecting the single best predictor” [35] and similar conclusions were drawn in van der Laan’s research [36]. Recently, Gelman’s team illustrated the theories about stacking for multimodal Bayesian posterior distributions and generalized the stacking to Bayesian hierarchical stacking, in which the weights of sub-models vary as a function of data, and are inferred using Bayesian inference [37, 38]. Despite the demonstrated advantages in prediction, Bayesian inference encounters challenges in intensive computation.

In this study, we proposed a novel stacking strategy for predicting disease risk using a non-negative spike-and-slab Lasso (nsslasso) generalized linear model (GLM) in the context of high-dimensional omics data. Precisely, we use the group structure information derived from biological knowledge to segment high-dimensional omics data into several sub-data. After that, each sub-model is trained separately using the features from the grouped feature set via a proper base learner (better prediction and shorter time cost) and a CV procedure. Then, the CV predictions of sub-models are ensembled by the super learner using nsslasso GLM. We propose several variants based on the above strategy by combining different base learners and super learners and assessing their prediction performance via a simulation study. These methods are also compared with several widely used penalized methods. Without loss of generality, the proposed methods are applied to large-scale gene expression data derived from two open-access breast cancer datasets using pathways as the group information.

The paper is organized as follows: In “Methods” section, we provide a detailed illustration of the stacking fitting procedure using the nsslasso GLM, along with the algorithm for parameter estimation using Expectation–Maximization (EM) and the cyclic coordinate descent algorithm. "Simulation study" section presents a comparison of the prediction performance of our proposed method and existing methods through a simulation study. In "Applications to real data" section, we apply the proposed methods to real-world data. Finally, "Discussion" section concludes the paper and addresses several critical issues related to our approach.

## Methods

### nsslasso GLM stacking model

Given a learning dataset $D=\{({y}_{n}, {{\varvec{x}}}_{n}), n = 1, 2, ..., N\}$, suppose a numerical or binary outcome variable *y* in terms of an input vector ***x***, that can be predicted by fitting *J* predictive models {${f}_{1}(x), {f}_{2}(x), ..., {f}_{J}(x)$} either based on different modeling methods (e.g., random forest, support vector machine, etc.) or using the subsets of input variables. Instead of selecting the single optimal predictive model, stacking was proposed to combine the predictions of the *J* models.

The general model stacking is a two-layer structure consisting of base learners and the super learner. The first layer randomly partitions the original training data *D*_0_ into *K* mutually exclusive and exhaustive subsets (folds) of (rough) equal size. The *k*^th^ fold is used as a validation set, $V(k)$, while the remaining folds are used as a training set, $T(-k)$, $k = 1, 2, ..., K$, to predict the outcomes in $V(k)$. The process is repeated for each fold, resulting in the prediction for all data $V$. For *J* candidate base learners, we can obtain the prediction ${V}_{j}, j=1, 2, \dots , J$ by repeating the above procedure. The whole process yields a matrix with columns being pooled CV prediction for different base learners. The second layer implements a super learner to fit the CV predictions from *J* base learners. The resulting coefficients are the estimated weights ${\widehat{w}}_{j}$ for the *j*th base learner, which is subsequently used to combine the *J* sub-models. Of note, sub-models to combine are the refit models (here, denoting ${f}_{j}(x)$) using the original data *D*_0_. The final prediction model in a new data *D*_1_ is given by,

1 $$y = \mathop \sum \limits_{j = 1}^{J} \hat{w}_{j} f_{j} \left( x \right){ }$$

The estimated weights ${\widehat{w}}_{j}$ are usually constrained to be non-negative to lower the variance of prediction while the sum-to-one constraint of ${\widehat{w}}_{j}$ proved to be generally unnecessary [35]. Optimization algorithms, such as non-negative least squares and the limited-memory BFGS method (L-BFGS), can be used to estimate the weights [39].

**Fig. 1:**
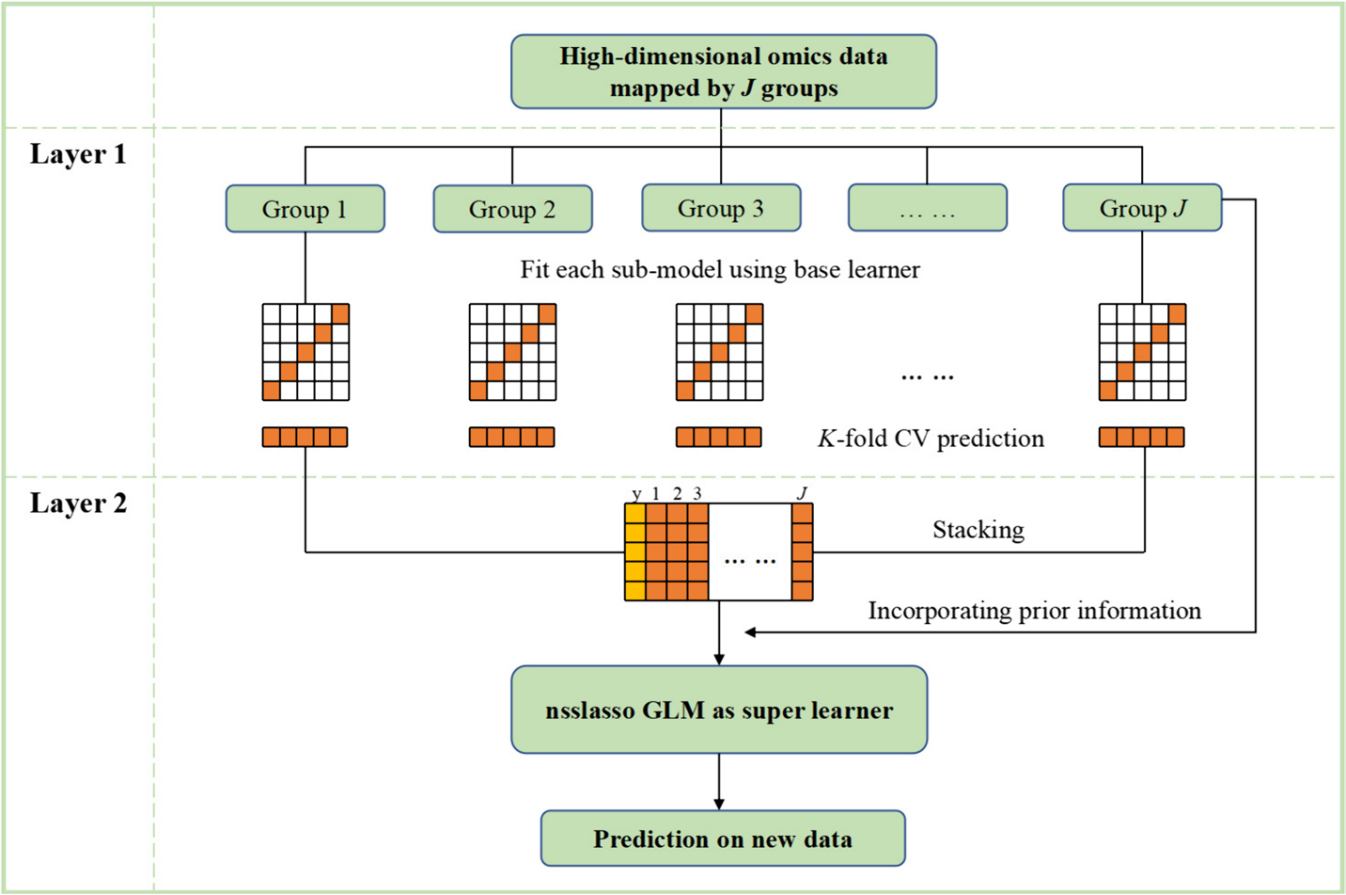
The algorithm flow plot of the nsslasso GLM stacking strategy based on segmenting high-dimensional omics data. GLM: generalized linear model; CV: cross-validation

In the present study, we introduced a novel nsslasso GLM stacking strategy based on segmenting high-dimensional omics data. The algorithm flow is shown in Fig. 1. High-dimensional omics data are segmented into groups based on prior biological knowledge. This reduces the dimensionality from considering all the variables to only considering those in a given group. Then we propose to construct predictive models based on features in each group, serving as sub-models of the first layer of the stacking framework. Sparse methods (SMs), such as the Lasso, SCAD, MCP, or network penalized method, as well as various machine learning (ML) methods, can be used to build sub-models. In the second layer, the nsslasso GLM is used as the super learner to estimate the weights of the sub-models based on the CV predictions. Here, we treat the predictions of the sub-models as covariates under a GLM. This idea has already been mentioned in [36, 40]. The final model can be expressed as,

2 $$h\left[ E \right.\left. {\left( y \right)} \right] = w_{0} + \mathop \sum \limits_{j = 1}^{J} \hat{w}_{j} f_{j} \left( x \right)$$

where the response variable *y* is supposed to follow an exponential family distribution; $h$ is a monotonic link function, such as an identity function or sigmoid function.

Here, we use an adaptive spike-and-slab mixture prior distribution $\psi \left(.\right)$ for weight ${\widehat{w}}_{j}$ in the super learner to differentiate sub-models according to their importance in predicting outcomes [41]. Let $\psi (.)$ follow the truncated mixture double-exponential (DE) prior distribution assuming that weights are restricted to be non-negative, (see Additional file 1: Fig. S1), then ${\widehat{w}}_{j}$ follows the non-negative spike-and-slab prior:

3 $$w_{j} |\gamma_{j} ,s_{0} ,s_{1} \sim \left( {1 - \gamma_{j} } \right)DE\left( {w_{j} |0,s_{0} } \right) + \gamma_{j} DE\left( {w_{j} |0,s_{1} } \right),{ }w_{j} \ge 0{ }$$

where *s*_0_ and *s*_1_ (*s*_1_ > *s*_0_ > 0) are the scale parameters for spike and slab distribution, respectively. *s*_1_ applies weaker compression to the pathways of strong effects and is usually given as a larger value, say *s*_1_ = 1; while *s*_0_ gives stronger compression to the pathways of weak effects (or even compress to zero) and is a smaller value, which should be selected from a set of predefined candidate values via cross-validation. ${\gamma }_{j}$ is an indicator (${\gamma }_{j} \in \{0, 1\}$) following a binomial distribution:

4 $$\gamma_{j} |\theta_{j} \sim {\text{Bin}}(\gamma_{j} |1,\theta_{j} ) = \theta_{j}^{{\gamma_{j} }} \left( {1 - \theta_{j} } \right)^{{1 - \gamma_{j} }}$$

where ${\theta }_{j}$ is a specific parameter for sub-models following the Beta distribution ${\theta }_{j}\sim Beta(a, b)$. This parameter can integrate external prior information. However, ${\theta }_{j}$ can reduce to ${\theta }_{j}\sim U(0, 1)$ in the absence of prior information. Formula (3) can further be represented as:

5 $$w_{j} |S_{j} \sim DE\left( {w_{j} |0,S_{j} } \right) = \frac{1}{{2S_{j} }}\exp \left( - \frac{{w_{j} }}{{S_{j} }}\right),{ }w_{j} \ge 0$$

where ${S}_{j}=(1-{\gamma }_{j}){s}_{0}+{\gamma }_{j}{s}_{1}$ is called the total scale parameter.

### Algorithm and parameters estimation

Parameters in the proposed model stacking framework including ${w}_{j}$, ${\theta }_{j}, {\gamma }_{j}$, and ${S}_{j}$ are estimated with the EM algorithm based on the cyclic coordinate descent algorithm instead of the Bayesian intensive sampling algorithm. This approach enables faster and more feasible fitting of high-dimensional models without compromising prediction accuracy.

In E-step, given ${w}_{j}$ and ${\theta }_{j}$, the posterior expectation of ${\gamma }_{j}$, denoting ${p}_{j}$, can be derived from,

6 $$\begin{aligned} p_{j} & = E(\gamma_{j} |w_{j} ,\theta_{j} ) = \mathop \sum \limits_{{\gamma_{j} \in \left( {0,{ }1} \right)}} \gamma_{j} p(\gamma_{j} |w_{j} ,\theta_{j} ) = p(\gamma_{j} = 1|w_{j} ,\theta_{j} ) \\ & = \frac{{p(w_{j} |\gamma_{j} = 1,{ }s_{1} )p(\gamma_{j} = 1|\theta_{j} )}}{{p(w_{j} |\gamma_{j} = 0,{ }s_{0} )p(\gamma_{j} = 0|\theta_{j} ) + p(w_{j} |\gamma_{j} = 1,{ }s_{1} )p(\gamma_{j} = 1|\theta_{j} )}} \\ \end{aligned}$$

where ${p(\gamma }_{j}=1|{\theta }_{j})={\theta }_{j}$, ${p(\gamma }_{j}=0|{\theta }_{j})=(1-{\theta }_{j})$, $p({w}_{j}|{\gamma }_{j}=1,{s}_{1})=\psi \left({w}_{j}|0,{s}_{1}\right)$, $p({w}_{j}|{\gamma }_{j}=0,{ s}_{0})=\psi \left({w}_{j}|0,{ s}_{0}\right)$. Then, denote the conditional posterior expectation of ${{S}_{j}}^{-1}$ as ${\lambda }_{j}$ and it can be derived from,

7 $$\begin{aligned} & \lambda_{j} = E(S_{j}^{ - 1} |w_{j} ,\theta_{j} ) = E\left( {\left[ {\left( {1 - \gamma_{j} } \right)s_{0} + \gamma_{j} s_{1} } \right]^{ - 1} |w_{j} ,\theta_{j} } \right) \\ & \quad = \left( {\frac{1}{{\left( {1 - \gamma_{j} } \right)s_{0} + \gamma_{j} s_{1} }}|\gamma_{j} = 0} \right)p(\gamma_{j} = 0|w_{j} ,\theta_{j} ) \\ & \quad + \left( {\frac{1}{{\left( {1 - \gamma_{j} } \right)s_{0} + \gamma_{j} s_{1} }}|\gamma_{j} = 1} \right)p(\gamma_{j} = 1|w_{j} ,\theta_{j} ) \\ & \quad = \left( {1 - p_{j} } \right)/s_{0} + p_{j} /s_{1} \\ \end{aligned}$$

In M-step, we update ($w$, $\theta$) by maximizing the log joint posterior density of these parameters,

8 $$\begin{aligned} & \log p(w,\theta |y,\gamma ,S) \propto l\left( w \right) - \mathop \sum \limits_{j = 1}^{J} w_{j} /S_{j} \\ & \quad + \mathop \sum \limits_{j \in J} \left[ {(\gamma_{j} \log \left( {\theta_{j} } \right) + \left( {1 - \gamma_{j} } \right)\log \left( {1 - \theta_{j} } \right)} \right) + \log p\left( {\theta_{j} } \right)] \\ \end{aligned}$$

where $l(w)=logp(y|wf(x))$ is the log joint density distribution function of the sub-models; $p({\theta }_{j})$ is the prior distribution form of ${\theta }_{j}$, say, ${\theta }_{j}\sim Beta(a, b)$.

The estimate of $w$ can be updated by the following likelihood function,

9 $$Q_{1} \left( w \right) = l\left( w \right) - \mathop \sum \limits_{j = 1}^{J} \lambda_{j} w_{j}$$

where ${\lambda }_{j}$ is replaced by its conditional posterior expectation derived above. Noticed that the term ${\sum }_{j=1}^{J}{\lambda }_{j}{w}_{j}$ serves as the adaptive *L*_1_ Lasso penalty, and thus the weights can be updated by maximizing ${Q}_{1}(w)$ using the cyclic coordinate descent algorithm. Therefore, this method is called spike-and-slab Lasso (sslasso) [41]. This step can be done with the help of the R package *glmnet*, and limits the estimate of $w$ to non-negative (that is nsslasso).

In addition, we adopted existing numerical optimization algorithms, such as L-BFGS to obtain $\theta$. The summarized algorithm flow refers to [41].

### Evaluation of model performance

The present study utilizes several metrics to measure the predictive performance of a fitted GLM, including (1) deviance: $-2{\sum }_{n=1}^{N}logp{(y}_{n}|wf(x))$; (2) the Brier Score (BS) for binary outcomes: $\frac{1}{N}\sum_{n=1}^{N}{{(y}_{n}-{\widehat{y}}_{n})}^{2}$; and (3) area under the ROC curve (AUC); (iv) misclassification for binary outcomes: $\frac{1}{N}\sum_{n=1}^{N}I(\left|{y}_{n}-{\widehat{y}}_{n}\right|>=0.5)$, where $I(\left|{y}_{n}-{\widehat{y}}_{n}\right|>0.5)=1$.

### Competitive statistical methods

We assess the prediction performance of the proposed approach using simulated and two real data. For the stacking methods, Lasso (*glmnet*) [42], SCAD, MCP (*ncvreg*) [43], and network penalized method (*glmgraph*) [9], as well as some ML methods including K-nearest neighbor (KNN), support vector machine (SVM), naive bayes (Nba), random forest (RF) (*E1071*) [44], are used as base learners. In addition to using nsslasso as the super learner (implemented with the package *BhGLM*, with the weights limited to non-negative) [45], we consider two competitive super learners, namely non-nagetive Lasso (nLasso) (implemented in the package *glmnet*, with the weights limited to non-negative) and L-BFGS (the function optim() in the package *stats*). We use *K*-fold CV with *K* = 5 for stacking to ensure computational feasibility [25, 39]. Several potential competitive statistical methods are included: Lasso, MCP, SCAD, and network regularized method. Several group-level penalization methods are also used for comparison, such as the “all in all out” methods including overlap (to deal with overlapping structures) group Lasso (grlasso), overlap group MCP (grMCP) and overlap group SCAD (grSCAD) (*grpregOverlap*) [46], and the “bi-level” methods including cMCP (*grpregOverlap*) and group spike-and-slab Lasso (gsslasso) GLM (*BhGLM*). Another “bi-level” method in *grpregOverlap*, say group exponential Lasso (GEL) is not used because of its poor performance in this study. All methods are executed using default parameters. All analyses are performed using R (4.1.3) software on *Dale T7920 INTEL Windows 10 Gold 5117 CPU @ 2.00GHz*.

## Simulation study

### Simulation design

The present study designs six scenarios with three gradient-distributed theoretical generalized R^2^ [47] and two sets of varied non-zero covariate coefficients ($\beta$) (see Table 1) to quantify the amount of information in a given data set. For each scenario, we generate two homogeneous datasets with equal sample sizes, one for training data *D*_0_ and the other for test data *D*_1_. To assess the performance of the methods, we conduct 100 duplicated runs and calculate the average results for comparison. The simulations are implemented using the R package *BhGLM*.

**Table 1:** The parameters of six different simulation scenarios (N = 500, M = 1000)

Scenarios	Non-zero coefficients								Correlation coefficient *r*	Residual variance $\sigma$	^a^Adjusted generalized *R*^2^
	Group 1			Group 5			Group 20				
	*β*_5_	*β*_20_	*β*_40_	*β*_210_	*β*_220_	*β*_240_	*β*_975_	*β*_995_			
1	0.80	− 0.70	1.00	− 0.90	− 0.80	0.90	− 1.00	0.70	0.60	1.60	0.50
2	0.80	− 0.70	1.00	− 0.90	− 0.80	0.90	− 1.00	0.70	0.60	2.60	0.25
3	0.80	− 0.70	1.00	− 0.90	− 0.80	0.90	− 1.00	0.70	0.60	4.50	0.10
4	0.80	− 0.30	1.40	− 0.90	− 0.80	0.90	− 1.50	0.20	0.60	1.80	0.50
5	0.80	− 0.30	1.40	− 0.90	− 0.80	0.90	− 1.50	0.20	0.60	3.10	0.25
6	0.80	− 0.30	1.40	− 0.90	− 0.80	0.90	− 1.50	0.20	0.60	5.50	0.10

The final adjusted generalized *R*^2^ is adjusted through $\sigma$

^a^Adjusted generalized *R*^2^ was obtained by fitting all variables (M = 1000) with the logistic regression model using a large sample (N = 20,000)

Specifically, for each dataset, we generate *N* = 500 samples, each with a binary response ${y}_{n}$ and *M* = 1000 continuous covariates${{\varvec{x}}}_{n}=\left({x}_{n,1}, {x}_{n,2}, .., {x}_{n,1000}\right)$, for$n=1, 2, \dots , N$. The vector ***x***_*n*_ is randomly sampled from the multivariate normal distribution i.e. ${{\varvec{x}}}_{n}\sim N(0,\Sigma )$, where $\Sigma \in {\mathbb{R}}^{1000\times 1000}$ is the variance–covariance matrix. We then group these covariates into 20 distinct groups, allowing for overlap between the groups (Additional file 2: Table S1). The correlation coefficient *r* within groups is 0.6, while the variables between groups are independent. The binary response ${y}_{n}$ is generated by dichotomizing a continuous intermediate response ${z}_{n}$ with 50% largest being “positive” (${y}_{n} = 1$) and the others being “negative” (${y}_{n} = 0$). ${z}_{n}$ follows a univariate normal distribution${z}_{n}\sim N({\mu }_{n}, {\sigma }^{2})$, where${\mu }_{n}={\beta }_{0}+{\sum }_{m=1}^{M}{x}_{nm}{\beta }_{m}$, with ${\beta }_{0}$ set to zero in this study. ${\sigma }^{2}$ denotes the residual variance, which is determined by fixing three theoretical generalized R^2^: 0.50, 0.25, and 0.10. We set a total of eight non-zero covariate coefficients with two types: the absolute values range between 0.7 to 1, and the other range from 0.2 to 1.5.

### Results of the simulation

#### Prediction performance

Tables 2 and 3 summarize the Brier Score and AUC of different methods under six simulation scenarios. Deviance, misclassification, and running time are shown in Additional file 2: Table S2. Among SMB methods, MCP and SCAD are representative methods that use coefficient-adaptive penalties. According to the simulation, their performance varied little compared to Lasso in the six scenarios. The network method also performed similarly to Lasso. The methods considering group structures, e.g., grlasso, grMCP, and grSCAD, did not exhibit advantages in prediction compared with these neglecting group structures. Only gsslasso is competitive across all scenarios.

**Table 2:** Prediction performance of various methods on Brier Score (mean (SD)) across six scenarios and 100 duplicated runs

	Scenario 1	Scenario 2	Scenario 3	Scenario 4	Scenario 5	Scenario 6
*Penalty and group penalty methods*						
Lasso	0.148 (0.010)	0.202 (0.009)	0.235 (0.006)	0.160 (0.009)	0.190 (0.010)	0.232 (0.006)
MCP	0.147 (0.011)	0.201 (0.010)	0.236 (0.007)	0.159 (0.010)	0.189 (0.012)	0.233 (0.007)
SCAD	0.146 (0.010)	0.201 (0.009)	0.236 (0.006)	0.159 (0.010)	0.189 (0.011)	0.232 (0.006)
network	0.165 (0.008)	0.202 (0.008)	0.237 (0.006)	0.156 (0.009)	0.193 (0.009)	0.232 (0.005)
gsslasso	0.145 (0.011)	0.201 (0.008)	0.235 (0.007)	0.156 (0.010)	0.188 (0.011)	0.232 (0.007)
grlasso	0.165 (0.008)	0.210 (0.006)	0.240 (0.005)	0.174 (0.008)	0.201 (0.007)	0.238 (0.005)
grMCP	0.179 (0.007)	0.213 (0.007)	0.240 (0.004)	0.184 (0.008)	0.205 (0.007)	0.238 (0.005)
grSCAD	0.168 (0.008)	0.210 (0.006)	0.240 (0.005)	0.176 (0.007)	0.201 (0.007)	0.238 (0.005)
cMCP	0.148 (0.012)	0.202 (0.010)	0.237 (0.008)	0.160 (0.012)	0.194 (0.011)	0.233 (0.007)
*Model stacking methods*^a^						
nsslasso (Lasso)	0.162 (0.009)	0.199 (0.008)	0.232 (0.007)	0.148 (0.010)	0.185 (0.010)	0.229 (0.007)
nsslasso (MCP)	0.159 (0.009)	–	–	–	–	0.229 (0.007)
nsslasso (SCAD)	0.159 (0.009)	–	–	–	–	0.228 (0.007)
nsslasso (network)	0.160 (0.008)	0.199 (0.008)	0.237 (0.006)	0.146 (0.010)	0.185 (0.010)	0.229 (0.007)
nsslasso (KNN)	0.190 (0.010)	0.219 (0.009)	0.244 (0.008)	0.179 (0.011)	0.208 (0.009)	0.240 (0.007)
nsslasso (NBa)	0.205 (0.009)	0.226 (0.008)	0.243 (0.005)	0.191 (0.009)	0.214 (0.008)	0.238 (0.005)
nsslasso (RF)	0.182 (0.009)	0.212 (0.008)	0.240 (0.006)	0.167 (0.011)	0.199 (0.009)	0.236 (0.006)
L-BFGS (Lasso)	0.176 (0.010)	0.206 (0.008)	0.234 (0.005)	0.167 (0.013)	0.196 (0.008)	0.232 (0.005)
nLasso (Lasso)	0.162 (0.009)	0.199 (0.008)	0.232 (0.006)	0.148 (0.010)	0.185 (0.010)	0.228 (0.006)

^a^The first column displays the super learner outside the bracket and the base learner inside. “–” means unanalyzed due to complexity in computation

**Table 3:** Prediction performance of various methods on AUC (mean (SD)) across six scenarios and 100 duplicated runs

	Scenario 1	Scenario 2	Scenario 3	Scenario 4	Scenario 5	Scenario 6
*Penalty and group penalty methods*						
Lasso	0.871 (0.018)	0.754 (0.023)	0.644 (0.029)	0.848 (0.018)	0.786 (0.024)	0.660 (0.028)
MCP	0.873 (0.018)	0.757 (0.023)	0.637 (0.032)	0.851 (0.019)	0.785 (0.027)	0.655 (0.028)
SCAD	0.873 (0.017)	0.756 (0.023)	0.640 (0.029)	0.851 (0.019)	0.786 (0.026)	0.660 (0.026)
network	0.849 (0.019)	0.755 (0.023)	0.634 (0.029)	0.867 (0.020)	0.780 (0.026)	0.658 (0.025)
gsslasso	0.875 (0.017)	0.758 (0.022)	0.644 (0.029)	0.855 (0.018)	0.788 (0.025)	0.659 (0.028)
grlasso	0.842 (0.018)	0.738 (0.023)	0.624 (0.028)	0.825 (0.020)	0.762 (0.024)	0.636 (0.028)
grMCP	0.815 (0.020)	0.724 (0.022)	0.620 (0.026)	0.801 (0.022)	0.748 (0.024)	0.631 (0.027)
grSCAD	0.851 (0.018)	0.738 (0.023)	0.624 (0.027)	0.832 (0.019)	0.765 (0.022)	0.635 (0.028)
cMCP	0.871 (0.019)	0.752 (0.025)	0.634 (0.034)	0.850 (0.022)	0.772 (0.027)	0.653 (0.030)
*Model stacking methods*^a^						
nsslasso (Lasso)	0.845 (0.017)	0.763 (0.020)	0.656 (0.026)	0.870 (0.017)	0.796 (0.021)	0.670 (0.026)
nsslasso (MCP)	0.850 (0.016)	–	–	–	–	0.670 (0.027)
nsslasso (SCAD)	0.850 (0.016)	–	–	–	–	0.671 (0.027)
nsslasso (network)	0.847 (0.016)	0.762 (0.020)	0.634 (0.029)	0.872 (0.018)	0.800 (0.022)	0.669 (0.025)
nsslasso (KNN)	0.785 (0.022)	0.706 (0.027)	0.597 (0.041)	0.809 (0.022)	0.738 (0.024)	0.617 (0.037)
nsslasso (NBa)	0.749 (0.024)	0.686 (0.029)	0.609 (0.029)	0.782 (0.022)	0.722 (0.024)	0.635 (0.025)
nsslasso (RF)	0.802 (0.019)	0.728 (0.023)	0.620 (0.032)	0.835 (0.021)	0.763 (0.023)	0.638 (0.026)
L-BFGS (Lasso)	0.845 (0.017)	0.765 (0.019)	0.659 (0.027)	0.869 (0.017)	0.796 (0.021)	0.673 (0.025)
nLasso (Lasso)	0.844 (0.017)	0.763 (0.020)	0.656 (0.026)	0.870 (0.017)	0.796 (0.021)	0.670 (0.025)

For the stacking methods, we considered obtaining the sub-models using the SMs including Lasso and network, as well as ML methods including KNN, NBa, and RF, while MCP, SCAD, and SVM were not considered because of their complexity in computation (MCP and SCAD only listed in Scenarios 1 and 6, SVM took more than 90 min for each duplicated run). We employed nsslasso as the super learner and compared it to nLasso and L-BFGS. The computational time of nsslasso was similar to nLasso, but much shorter than L-BFGS. In our study, the ML-based stacking methods had poorer predictive performance compared to the SMB methods while the SMs-based stacking methods demonstrated a predictive advantage (except for Scenario 1) over the SMB methods. However, there was little difference between SMs-based stacking methods using different super learners.

#### Distribution of estimated weights

**Fig. 2:**
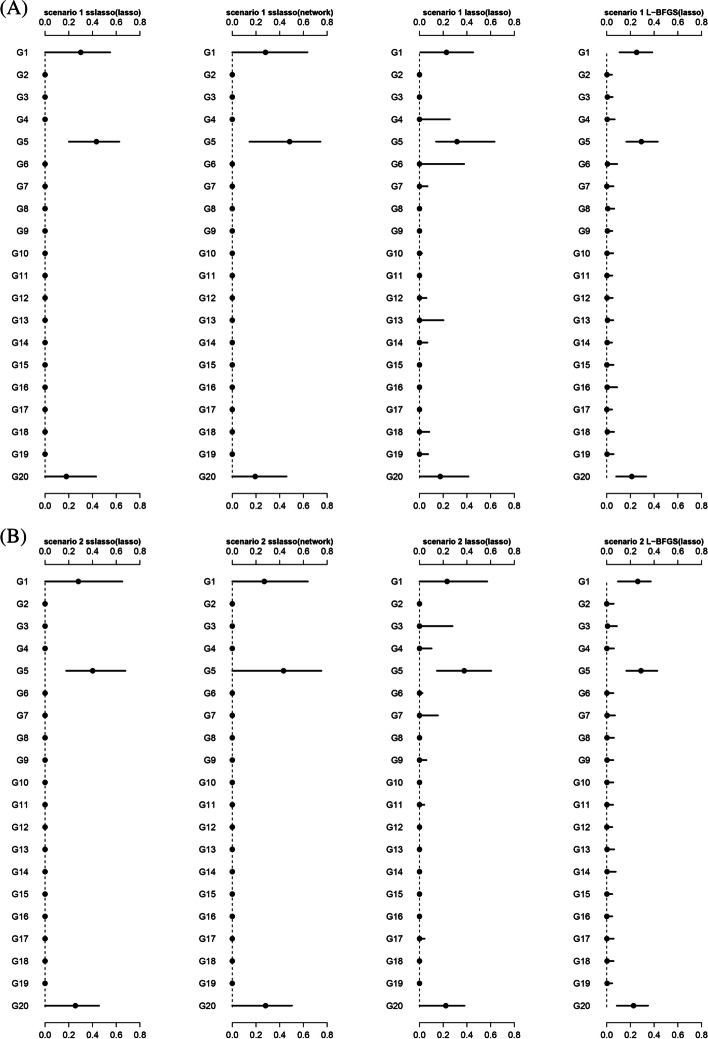

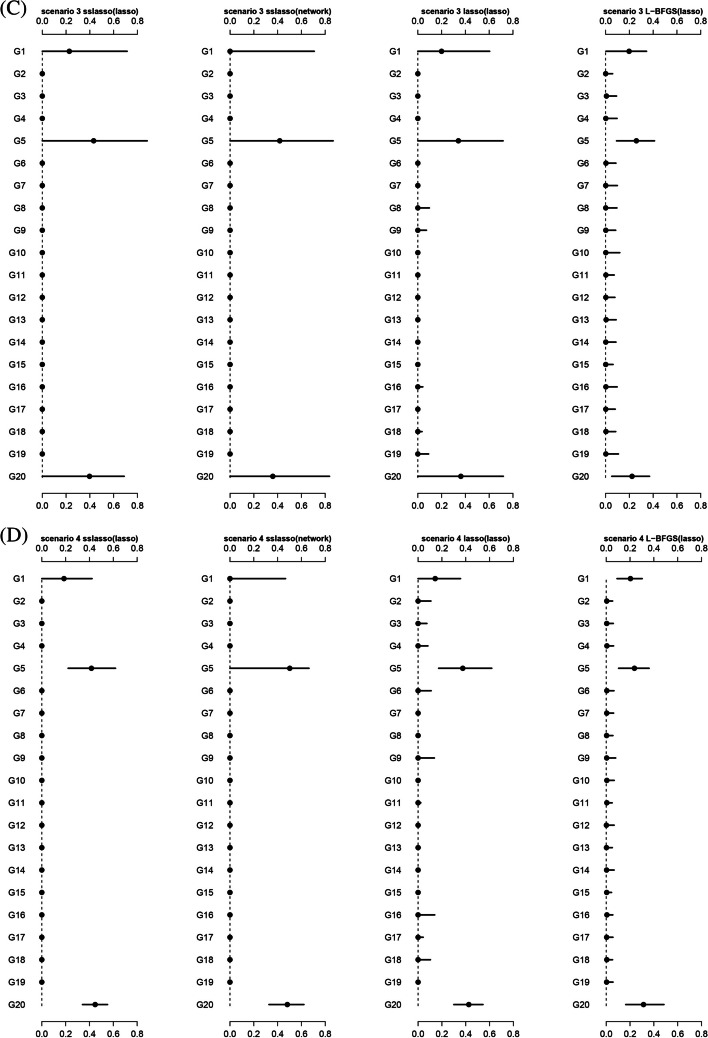

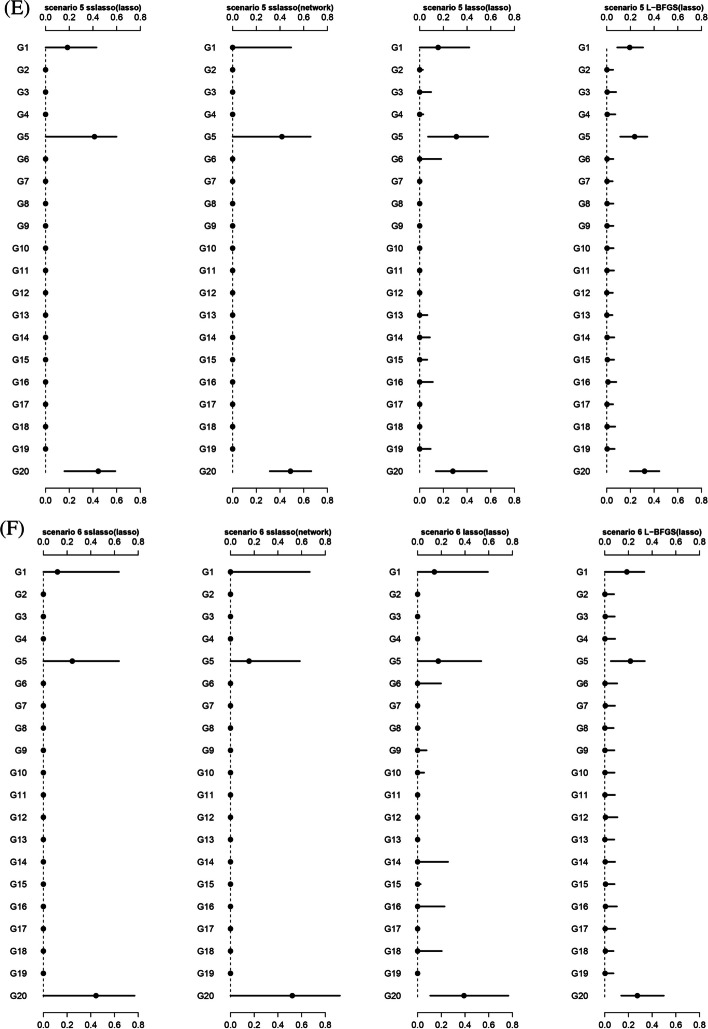
The distribution of weights estimated by model stacking methods in different scenarios. **A** Scenario 1; **B** Scenario 2; **C** Scenario 3; **D** Scenario 4; **E** Scenario 5; **F** Scenario 6. The estimated weights are normalized. The black dot represents the median and the line represents the 5–95 quantile interval

We further compared the weight estimations via SMs-based stacking methods using different super learners. Theoretically, the weights for group1, group5, and group20 should be non-zero due to the presence of relevant non-zero variables. Figure 2 shows that nsslasso consistently identified the non-zero weights across all scenarios, while L-BFGS and nLasso generally included some zero weights. Besides, L-BFGS had a narrower interval range of non-zero weights, but it may not be suitable for dealing with large amounts of sub-models because it lacks sparsity.

## Applications to real data

We applied the proposed approach to two real breast cancer datasets with binary outcomes and large-scale gene expression profiles. Breast cancer is the second leading cause of mortality in women, which is a typical molecular heterogeneous disease [48]. For these two datasets, gene expression data were standardized using the *covariates* function of *BhGLM* package in the R platform. We randomly partitioned the original data into two subsets of equal sample size: one for training models and the other for evaluating model performance. The process was repeated 100 times in case of casual results due to data split [49]. To ensure a balanced response, we performed a Chi-square test on the number of events between training and test data and considered those with ${P}_{chi-square}>0.2$ being balanced splits that would be retained for further analysis. Genes were mapped to pathways using genome annotation tools. More precisely, we first mapped gene symbols to Entrez Ids using *annotate* package and then mapped all genes to KEGG pathways (default parameter) using *clusterProfiler* package [50]. The adjacency matrix for each pathway used in the network regularization method was calculated using *WGCNA* package [51].

### TCGA breast cancer dataset

The Cancer Genome Atlas (TCGA) project collects a variety of types of cancer data such as clinical data, transcriptome expression data, and genomic variation data. We acquired the transcriptome profiling data of the Breast Invasive Carcinoma (BRCA) and the phenotype information from the “GDC Data Portal”. The outcome used in the present study is the occurrence of the “new tumor event”.

We included the samples that had both phenotype and expression profiles. Genes with > 50% of zero expression were filtered out, and those with > 20% quantile variance were kept. Eventually, we obtained a dataset with 960 samples and 14,068 genes. These genes were mapped to 162 pathways involving 4169 non-overlapping genes (see Additional file 2: Table S3). The study first conducted an initial screening of 162 pathways to identify those with potential predictive values. We fitted Lasso Logistic regression for these genes in each pathway and obtained the CV predicted values and AUC. From 129 pathways with AUC > 0.500, 19 pathways (1570 non-overlapping genes, Additional file 2: Table S3) with AUC > 0.577 were selected as candidate sub-models for subsequent analysis. For methods without considering group structures, models were directly fitted using the 1570 genes.

Table 4 summarizes the prediction performance of different methods for the TCGA BRCA dataset. gsslasso exhibited the best predictive performance among the SMB methods. The ML-based stacking methods showed poor predictive performance while nsslasso (Lasso) and L-BFGS (Lasso) outperformed the SMB methods according to AUC. In addition, we repeated the above analysis using 51 pathways with AUC > 0.550 (including a total of 2734 non-overlapping genes) to evaluate the impacts of the number of included pathways (see Additional file 2: Table S4). In general, nsslasso (Lasso) and L-BFGS (Lasso) still outperformed the other methods, although all methods experienced a decline in predictive accuracy.

**Table 4:** TCGA breast cancer data (N = 960, events = 196). The prediction performance of stacking and the other methods (mean (SD)). Results are based on 100 random splits of the original data to training set (N = 480) and test set (N = 480) (19 candidate pathways)

	Deviance	AUC	Brier score	Misclassification
*Penalty and group penalty methods*				
Lasso	479.216 (20.363)	0.587 (0.037)	0.160 (0.008)	0.203 (0.014)
MCP	481.482 (23.224)	0.575 (0.045)	0.161 (0.009)	0.203 (0.015)
SCAD	481.901 (26.133)	0.586 (0.040)	0.160 (0.008)	0.203 (0.014)
gsslasso	479.552 (21.890)	0.594 (0.031)	0.160 (0.009)	0.203 (0.013)
grlasso	481.971 (18.018)	0.554 (0.035)	0.161 (0.008)	0.203 (0.013)
grMCP	487.903 (26.992)	0.537 (0.033)	0.163 (0.009)	0.204 (0.015)
grSCAD	481.545 (18.039)	0.559 (0.034)	0.161 (0.008)	0.202 (0.014)
cMCP	497.776 (49.826)	0.577 (0.038)	0.165 (0.013)	0.210 (0.021)
*Model stacking methods*				
nsslasso (Lasso)	481.597 (20.492)	0.598 (0.027)	0.161 (0.008)	0.207 (0.013)
nsslasso (network)	485.152 (16.481)	0.583 (0.010)	0.163 (0.007)	0.208 (0.011)
nsslasso (KNN)	488.225 (21.950)	0.549 (0.035)	0.163 (0.009)	0.204 (0.013)
nsslasso (NBa)	489.239 (19.413)	0.531 (0.022)	0.163 (0.008)	0.202 (0.014)
nsslasso (RF)	486.269 (20.569)	0.551 (0.032)	0.162 (0.008)	0.203 (0.013)
L-BFGS (Lasso)	476.821 (18.592)	0.602 (0.026)	0.159 (0.008)	0.202 (0.013)
nLasso (Lasso)	480.495 (18.962)	0.583 (0.042)	0.161 (0.008)	0.205 (0.013)

In searching for model interpretation, we applied nsslasso (Lasso) to the whole data, resulting in a pathway-stacking model (AUC: 0.750) that selected five pathways fitted with a limited number of genes: p53 signaling pathway (hsa 04115, relatively weight, W = 0.084), RNA transport (hsa 03013, W = 0.297), Terpenoid backbone biosynthesis (hsa 00900, W = 0.215), RNA degradation (hsa 03018, W = 0.232), Arrhythmogenic right ventricular cardiomyopathy (hsa 05412, W = 0.172). All five pathways were included in the 19 selected pathways with AUC > 0.577. In addition, we also applied nLasso (Lasso) (AUC: 0.736) and L-BFGS (Lasso) (AUC: 0.746) for reference (see Additional file 2: Table S5). nLasso (Lasso) identified seven pathways, all of which were also selected by nsslasso (Lasso). L-BFGS (Lasso) included 57 pathways with relative weight > 0.001, which makes it difficult to indicate pathway importance.

### METABRIC dataset

The Molecular Taxonomy of Breast Cancer International Consortium (METABRIC) dataset encompasses over 2000 breast cancer patients with accessible clinical, gene expression, and mutation data. We acquired gene expression data and the phenotype data of the breast invasive ductal carcinoma from the METABRIC. The interested outcome is the “vital status”. After data preprocessing (as described in 4.1), we finally obtained a dataset with 1420 samples and 19,494 genes. These genes were mapped to 146 pathways involving 3709 non-overlapping genes (see Additional file 2: Table S6). We selected 21 pathways (Additional file 2: Table S6) with AUC > 0.669 as candidate sub-models for subsequent analysis.

In the SMB methods, grlasso and grSCAD were two competitive methods (Table 5). But these two methods did not perform well when applied to the TCGA BRCA dataset. The ML-based stacking methods also showed poor performance while the lasso-based super learners showed comparable performance to grlasso and grSCAD. We included 143 from 146 pathways with AUC > 0.600 (a total of 3,673 non-overlapping genes) as sensitivity analysis (Additional file 2: Table S7). Also, the performance of all methods decreased, and lasso-based super learners demonstrated favorable performance.

**Table 5:** METABRIC data (N = 1420, events = 621)

	Deviance	AUC	BS	Misclassification
*Penalty and group penalty methods*				
Lasso	909.438 (11.110)	0.677 (0.015)	0.225 (0.004)	0.374 (0.015)
MCP	921.284 (13.683)	0.662 (0.016)	0.229 (0.004)	0.385 (0.017)
SCAD	910.031 (10.286)	0.676 (0.014)	0.225 (0.003)	0.376 (0.015)
gsslasso	906.799 (12.149)	0.678 (0.014)	0.224 (0.004)	0.371 (0.014)
grlasso	909.698 (9.553)	0.682 (0.016)	0.225 (0.003)	0.368 (0.014)
grMCP	935.226 (146.534)	0.664 (0.020)	0.229 (0.012)	0.378 (0.017)
grSCAD	909.427 (10.034)	0.682 (0.016)	0.225 (0.003)	0.368 (0.015)
cMCP	951.428 (155.631)	0.662 (0.019)	0.233 (0.016)	0.381 (0.017)
*Model stacking methods*				
nsslasso (Lasso)	907.198 (15.916)	0.683 (0.015)	0.224 (0.005)	0.369 (0.015)
nsslasso (network)	908.390 (15.794)	0.681 (0.015)	0.224 (0.005)	0.371 (0.014)
nsslasso (KNN)	951.428 (17.056)	0.660 (0.018)	0.229 (0.005)	0.383 (0.016)
nsslasso (NBa)	928.802 (13.804)	0.655 (0.016)	0.231 (0.005)	0.385 (0.015)
nsslasso (RF)	901.347 (12.669)	0.681 (0.014)	0.222 (0.004)	0.371 (0.013)
L-BFGS (Lasso)	905.644 (7.404)	0.688 (0.014)	0.223 (0.003)	0.371 (0.015)
nLasso (Lasso)	904.968 (14.092)	0.684 (0.015)	0.223 (0.004)	0.369 (0.014)

The prediction performance of stacking and the other methods (mean (SD)). Results are based on 100 random splits of the original data to training set (N = 710) and test set (N = 710) (21 candidate pathways)

The pathway-stacking model fitted using nsslasso (Lasso) for the METABRIC dataset identified eight pathways (AUC: 0.777): cell cycle (hsa 04110, W = 0.174), HTLV-I infection (hsa 05166, W = 0.105), Calcium signaling pathway (hsa 04020, W = 0.166), Protein digestion and absorption (hsa 04974, W = 0.092), Adipocytokine signaling pathway (hsa 04920, W = 0.102), PPAR signaling pathway (hsa 03320, W = 0.086), TGF-beta signaling pathway (hsa 04350, W = 0.156), Protein processing in the endoplasmic reticulum (hsa 04141, W = 0.119). nLasso (Lasso) (AUC: 0.776) covered nsslasso (Lasso) (Additional file 2: Table S8). L-BFGS (Lasso) (AUC: 0.760) identified 82 pathways with relatively small weights (> 0.001).

## Discussion

The present study proposed a general stacking strategy based on data segmentation and nsslasso GLM for predicting disease risk in the context of high-dimensional data. To the best of our knowledge, this is the first paper that demonstrates the use of stacking to integrate group structure information into modeling, in which a new non-negative spike-and-slab prior that limits the weights of sub-models to non-negative is used. The proposed method inherits the advantage of stacking, which may account for the improved and robust generalization compared to those existing methods based on the single model (e.g., Lasso, grlasso, gsslasso). Furthermore, employing nsslasso as the super learner can adaptively combine sub-models: selecting a strong sub-model but eliminating the rest with similar effects. This feature leads to reduced variance and enhanced prediction accuracy [35]. Using nsslasso is comparable to using L-BFGS in prediction, but the former exhibits an advantage in fast estimating weights and identifying important sub-models.

In the simulation, the SMs-based methods exhibited superior performance in prediction than the SMB methods, except for Scenario 1. Scenario 1 represented the situation of high theoretical generalized R^2^ (low ${\sigma }^{2}$), in which the data noise is low. In the case of enough effective information, methods based on a single model can achieve a fairly good prediction. Besides, stacking methods suffer from an increased variance due to the random split in the CV procedure [52], which can potentially lead to the loss of valuable information. With the increase of data noise (much closer to real-world data) and the decrease of effective information, the SMs-based stacking methods presented a better performance in simulation scenarios and real-world data, because it is more tolerant to noise by borrowing information from different models.

For this study, we conducted 100 duplicated runs for every scenario. To evaluate the impact of the number of duplicated runs, we increased the simulation runs to 200 for Scenario 1 and compared the results with those obtained from 100 runs. The stacking methods remained consistent across both 100 and 200 runs, while the SMB showed slight changes. This suggests that the stacking-based methods are more stable in making predictions.

In addition, the ML-based stacking methods showed poor prediction performance either in simulated data or in real-world data. One possible explanation is that these ML methods are prone to overfit the data. These overfited sub-models, typically, produce similar predictions. Stacking’s performance is likely to be less favorable when the sub-models yield similar predictions. Another possible reason is that these ML methods with complex fitting algorithms are generally less appropriate for the data of a small sample size. These methods require more data to fit their parameters well [25].

A noted point of the proposed strategy is the interpretability of the resulting models. As stated in Buch’s article [11], the utilization of prior biological knowledge for the purpose of grouping omics data can identify relevant functional groups. In our study, for instance, five important pathways were identified by nsslasso (Lasso) in TCGA breast cancer concerning the occurrence of “new tumor event”, out of which the p53 signaling pathway is one of the most well-known pathways that is closely associated with the prognosis of breast cancer [53]. The aberrant of p53 results in an elevated occurrence of new tumor events as many signals about cellular health interact with the p53 protein, ultimately determining whether the cell proceeds with the division cycle [54]. The model also identified other pathways that involve various biological processes. RNA transport (weight: 0.297), RNA degradation (weight: 0.232), and Terpenoid backbone biosynthesis (weight: 0.215) contributed more to the prediction, highlighting their important role in prediction. Meanwhile, the proposed stacking strategy carries out a within-group variable selection to extract genes at the base layer. With the help of power from genes in the same pathway, one may identify patterns that are too subtle to discern at the single gene level [11, 22]. These identified pathways and genes can serve as starting points for subsequent targeting research.

Variable screening is essential when the dimensionality of the data is extremely large [25]. The proposed methods involve at least three dimension reduction processes, which may account for the observed favorable performance. The first dimension reduction is the data segmentation based on prior biological information, whereby, reducing the whole omics data to a set of sub-data. The second dimension reduction involves the variable selection using sparse methods in the construction of sub-models, reducing sub-data to several important predictors. The third dimension reduction is the selection of important sub-models using the proposed nsslasso GLM in the second layer of stacking. This sequence of dimension reductions gradually eliminates numerous irrelevant variables, ensuring that the stacked models contain only a limited number of vital predictors.

A notable challenge of our approach is its computationally intensive nature, primarily due to the CV process and the need to ensemble numerous sub-models. Therefore, our practice is to first select the pathways with strong signals (say, twenty pathways of the highest AUC) as candidate sub-models. Moreover, as a hierarchical Bayesian stacking method, our method can be extended by incorporating multiple-level group structures, such as SNP-gene-pathway. This extension can be achieved by leveraging prior knowledge of $\theta$. In addition, researchers can explore alternative priors to the non-negative spike-and-slab mixture DE prior used in the proposed model and investigate their theoretical and empirical properties. Last but not least, the proposed method is a common strategy, which can be applied to other biological processes with similarly multiple levels.

### Supplementary Information

**Additional file 1**. Supplementary figures.

**Additional file 2**. Supplementary tables.

## Data Availability

The main code for the proposed method is freely available on the GitHub website at: (https://github.com/JasonLnzi/A-Non-negative-Spike-and-slab-Lasso-Generalized-Linear-Stacking-Model). The R package and function mentioned in the section “2.4 Competitive statistical methods” are listed in Additional file 2: Table S10. We acquired the dataset for Breast Invasive Carcinoma (ldentifier/Accession Number: TCGA-BRCA) from the TCGA (The Cancer Genome Atlas) database, accessible at https://portal.gdc.cancer.gov/projects/TCGA-BRCA. We obtained another breast cancer data from METABRIC (Molecular Taxonomy of Breast Cancer International Consortium, https://www.cbioportal.org/study/summary?id=brca_metabric) with the identifier “Breast Invasive Ductal Carcinoma”.

## Abbreviations

Lasso: Least absolute shrinkage and selection operator
nsslasso: Non-negative spike-and-slab lasso
GLM: Generalized linear model
SCAD: Smoothly clipped absolute deviation
MCP: Minimax concave penalty
grlasso: Group lasso
grSCAD: Group SCAD
cMCP: Composite MCP
MLGL: Multi-layer group-lasso
OHPL: Ordered homogeneity pursuit lasso
SMB: Single-model-based
CV: Cross-validation
EM: Expectation-maximization
L-BFGS: Smoothly clipped absolute deviation
SMs: Sparse methods
ML: Machine learning
DE: Double-exponential
sslasso: Spike-and-slab lasso
BS: Brier Score
ROC: Receiver operating characteristic curve
AUC: Area under the ROC curve
grMCP: Overlap group MCP
GEL: Group exponential lasso
KEGG: Kyoto encyclopedia of genes and genomes
TCGA: The Cancer Genome Atlas
BRCA: Breast cancer
METABRIC: Molecular Taxonomy of Breast Cancer International Consortium
