# Supplementary material for: A non-negative spike-and-slab lasso generalized linear stacking prediction modeling method for high-dimensional omics data
Source: BMC Bioinformatics. 2024 Mar 20;25:119. doi: 10.1186/s12859-024-05741-6 (PMC10953151; doi:10.1186/s12859-024-05741-6)
Supplement: Supplementary file 1 — Additional file 1. Supplementary figures. [file 12859_2024_5741_MOESM1_ESM.docx]

CONTENT

**Supplementary figures**

Supplementary Figure 1. Diagram of non-negative spike-and-slab mixed prior distribution 1

**
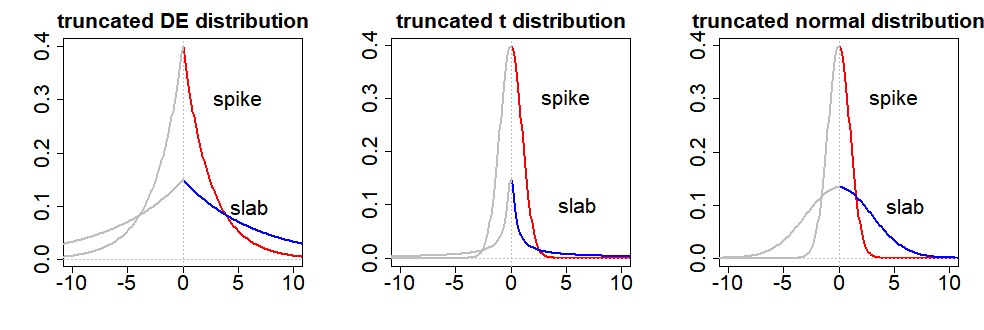
**

**Figure S1: Diagram of non-negative spike-and-slab mixed prior distribution.** The left panel is a truncated DE distribution with obvious peaks and gentle slop compared to truncated t distribution (the middle panel) and truncated normal distribution (the right panel).

# Abbreviations used in the Supplementary Figures

DE: double-exponential
